# NiTi–Cu Bimetallic Structure Fabrication through Wire Arc Additive Manufacturing

**DOI:** 10.3390/ma17051006

**Published:** 2024-02-22

**Authors:** Shalini Singh, Elena Demidova, Natalia Resnina, Sergey Belyaev, Palani Anand Iyamperumal, Christ Prakash Paul, Konda Gokuldoss Prashanth

**Affiliations:** 1 Mechatronics and Instrumentation Laboratory, Discipline of Mechanical Engineering, Indian Institute of Technology Indore, Indore 453552, Madhya Pradesh, India; s13singh2013@gmail.com (S.S.); palaniia@iiti.ac.in (P.A.I.); 2 Department of General Mathematics & Informatics, Saint-Petersburg State University, Saint-Petersburg 199034, Russia; lena-demi@yandex.ru (E.D.); resnat@mail.ru (N.R.); spbelyaev@mail.ru (S.B.); 3 Raja Ramanna Centre for Advanced Technology, Indore 452013, Madhya Pradesh, India; drcppaul2015@gmail.com; 4 Homi Bhabha National Institute, Anushaktinagar, Mumbai 400094, Maharashtra, India; 5 Department of Mechanical and Industrial Engineering, Tallinn University of Technology, 19086 Tallinn, Estonia; 6 Centre for Biomaterials, Cellular and Molecular Theranostics (CBCMT), Vellore Institute of Technology, Vellore 632014, Tamil Nadu, India

**Keywords:** structure, shape memory alloys, wire arc additive manufacturing

## Abstract

This study endeavors to comprehensively explore and elucidate the seamless integration of NiTi shape memory alloys (SMAs) into multifaceted applications through the utilization of novel joining techniques. The primary focus lies in the utilization of wire arc additive manufacturing (WAAM) to deposit Nitinol (NiTi) onto Copper (Cu), thereby introducing a transformative approach for their integration into electro-mechanical systems and beyond. Through a detailed examination of the NiTi/Cu bimetallic junction, using advanced analytical techniques including SEM, XRD, and DSC analyses, this research aims to unravel the intricate complexities inherent within the interface. The SEM images and X-ray patterns obtained reveal a complex and nuanced interface characterized by a broad mixed zone comprising various constituents, including Ti(Ni,Cu)_2_, pure Cu, Ti_2_(Ni,Cu)_3_ precipitates, and Ni-rich NiTi precipitates. The DSC results, showcasing low-intensity broad peaks during thermal cycling, underscore the inherent challenges in demonstrating functional properties within the NiTi/Cu system. Recognizing the critical importance of an enhanced martensitic transformation, this study delves into the effects of heat treatment. Calorimetric curves post-annealing at 500 °C exhibit distinct transformation peaks, shedding light on the intricate influence of NiTi layer distribution within the junction. The optimal heat treatment parameters for NiTi/Cu junction restoration are meticulously explored and determined at 500 °C for a duration of 12 h. Furthermore, the study offers valuable insights into optimizing NiTi–Cu joints, with micro-hardness values reaching 485 HV and compressive strength scaling up to 650 MPa. These significant findings not only hold promise for diverse applications across various industries but also pave the way for further research directions and explorations into the realm of SMA integration and advanced joining methodologies.

## 1. Introduction

In recent years, there has been a noticeable uptick in the utilization of NiTi Shape Memory Alloys (SMAs) across a wide spectrum of industrial and biomedical applications [1,2,3,4,5]. The successful integration of NiTi hinges not only on leveraging the material’s inherent properties but also on resolving application-specific challenges, such as advancing joining methodologies. Developing a more robust technique is imperative for high-strength actuators [6,7] and achieving this could be facilitated through the metallurgical bonding of NiTi with other materials [8,9,10]. Hybrid NiTi systems incorporating diverse cost-effective materials hold promise for enabling novel functionalities and reducing manufacturing costs [9,10,11,12].

Researchers have dedicated considerable efforts to identifying the optimal welding parameters conducive to exploiting the shape memory alloy (SMA) properties, with particular emphasis on achieving defect-free joints. This endeavor aligns with the broader goal of harnessing SMA capabilities in welding applications [13]. Furthermore, ongoing investigations delve into the intricate metallurgical joining mechanisms occurring at the interface of materials during the ultrasonic spot welding of NiTi shape memory alloys with a Cu interlayer. This facet of research seeks to elucidate the nuanced interplay of materials at the microstructural level, shedding light on the mechanisms underpinning successful welding processes involving these specific alloys [14]. The hybrid system comprising NiTi and conventional engineering alloys, such as Cu-based alloys and steel, holds considerable advantages for various industries due to their functional features like shape memory effect and superelasticity. Copper-based alloys exhibit distinct properties such as corrosion resistance, ductility, good thermal conductivity, and excellent electrical conductivity [15,16,17,18]. However, due to NiTi’s susceptibility to high temperatures and the alteration of its shape memory effect and superelasticity in the weld zone compared to the base metal, due to induced metallurgical modifications and especially in dissimilar combinations, joining NiTi poses significant challenges.

Several welding techniques have been explored for NiTi SMAs, including resistance and arc welding [19,20], friction stir welding [21], and laser welding [9,10,11,12,22,23]. Laser and explosive welding are the most popular joining techniques for NiTi SMAs. Laser welding methods offer advantages for joining materials with low weldability, like NiTi, owing to features such as precision, high energy density, minimal heat-affected zones, low residual strains, fusion zones, and minimal distortion. However, the unique equipment and explosive requirements of explosive welding make large-scale production challenging. Laser welding and explosive welding are limited to joining plate and sheet samples, hindering the production of components with complex shapes. The procedures used to fabricate multi-material components often cannot produce complex-shaped components due to inherent constraints; hence, additive manufacturing (AM) is being extensively probed for various industrial applications [24,25,26]. Among the advanced manufacturing techniques, AM can fabricate components with accuracy and with any distinct shapes. Wire Arc Additive Manufacturing (WAAM) offers numerous advantages over conventional metal AM processes, including high deposition rates, cost-effectiveness, design flexibility, and high material utilization efficiency [27,28,29,30,31,32,33,34,35,36]. WAAM melts wire feedstock and deposits the material layer by layer to create complex 3D structures. WAAM finds its applications in various industries such as aviation, the maritime and automotive industries, and architecture. Since it utilizes wire-form raw materials and can deposit wires (welding), WAAM can process a variety of metals. However, complex-shaped NiTi and Cu components are challenging to manufacture using traditional methods such as turning, casting, milling, and forging [28,29]. Compared to the widely utilized fusion-based AM techniques like Electron Beam Melting and Selective Laser Melting [30,31,32], the WAAM fabrication of NiTi structures has proven effective in producing parts at reduced costs and with high deposition rates [33,34]. Creating dissimilar junctions between NiTi and Cu-based materials is relatively challenging due to the significant variations in their chemical and physical properties [34]. Published reports focus on a thorough analysis of functional fatigue in NiTi–Cu dissimilar welded joints, emphasizing the influence of ND: the YAG laser welding parameters on the microstructure and joint break force. Laser welding emerges as the primary choice among various joining technologies for NiTi alloys. It has been established that intermetallic phases like CuTi, Cu_4_Ti_3_, and Cu_2_Ti, which are brittle, render the welded connection more prone to brittleness [34,35]. The primary objective of this work is to fabricate NiTi–Cu joints through Wire Arc Additive Manufacturing (WAAM) and subsequently comprehensively characterize them. The study encompasses experimental techniques, parameter optimization, and in-depth analyses of the microstructures, phase transformations, and mechanical properties exhibited by the joints. Microstructural studies, including microscopy and X-ray diffraction (XRD) analysis, play a pivotal role in understanding the intricate details of the NiTi–Cu joints. These analyses offer valuable insights into the grain structure, phase composition, and crystallographic orientation within the joint interface, thereby aiding in the assessment of the overall integrity and performance of the welded assemblies. The goal is to provide insights that contribute to understanding the functional behavior of the NiTi/Cu assembly, considering both macroscopic and microscopic aspects. This includes exploring their mechanical capabilities for traction in large-scale structures and evaluating their performance under stress-loaded conditions.

Additionally, the established connecting technique holds promise for innovative applications of Shape Memory Alloys (SMAs), offering potential advancements and improvements in various engineering applications. In conclusion, the utilization of NiTi SMAs in diverse industries is poised for substantial growth, driven by ongoing research and advancements in joining methodologies and additive manufacturing techniques. The primary objective of this work is to fabricate NiTi–Cu joints through Wire Arc Additive Manufacturing (WAAM) and subsequently comprehensively characterize them.

## 2. Materials and Methods

**Figure 1:**
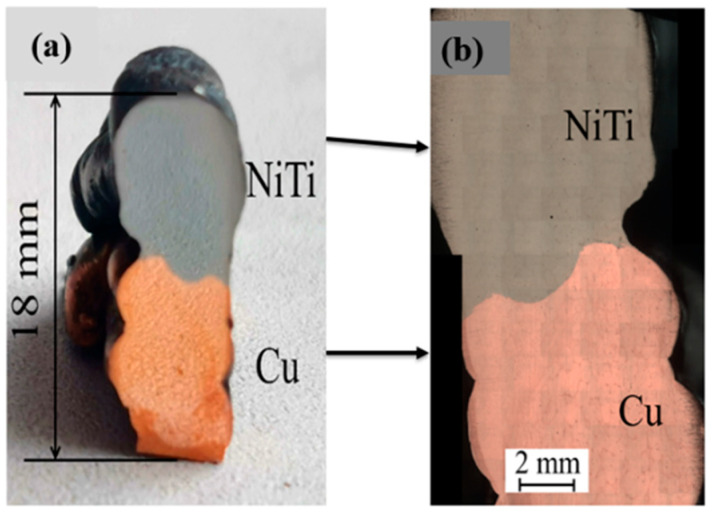
Photos (**a**) and images obtained with an optical microscope (**b**) of the NiTi/Cu bimetallic junction, produced by WAAM.

In this comprehensive study, Nitinol (Ni51Ti49) and copper wire with a diameter of 1.2 mm were carefully selected as feedstock materials. The substrate, comprising a mild steel plate measuring 100 mm × 50 mm × 8 mm, formed the foundation for the investigation. Utilizing a gas metal arc welding-based WAAM process, the research aimed to fabricate bimetallic wall structures seamlessly integrating NiTi and copper. A wire feeder unit, intricately connected to an X-Y stage, facilitated the delivery of the feedstock wire to the welding torch, while an electrode mounted on the X-Y stage allowed for multi-directional movement. The X-Y controller programmed using G and M code with Repitier host software Version 1 (Willich, Germany), played a crucial role in orchestrating the welding process. To ensure uniform deposition of Nitinol and copper and optimize structural consistency, optimized parameter selection for WAAM, as outlined in Table 1, was conducted. These adjustments addressed variations in width arising from the distinct thermo-physical properties of NiTi and Cu. A NiTi preheating temperature of 200 °C was carefully chosen for Cu deposition in WAAM to promote material bonding, minimize thermal stress, and prevent intermetallic compound formation, thereby enhancing joint strength and compatibility. This temperature, determined through extensive experimental trials, considered factors such as surface activation, reduced thermal stress, and potential interdiffusion. Preheating at the identified temperature not only fosters enhanced bonding but also facilitates a robust metallurgical bond between NiTi and Cu, ensuring the structural integrity of the joint. Additional details regarding the experimental setup are provided in a previously published report [36]. The joint assembly comprises five layers of NiTi and five layers of Cu, as depicted in Figure 1b. The observed irregularities in surface and shape can be attributed to the inherent challenges associated with effectively joining diverse materials through thermal processes [14]. This comprehensive exploration sheds invaluable light on the intricacies of bimetallic wall structure fabrication, paving the way for future advancements in material science and engineering.

Bimetallic junction NiTi/Cu produced by WAAM was cut perpendicular to deposition direction and polished according to standard procedure. Images of the polished samples, as well as microscopic view of the polished surface, are presented in Figure 1. After deposition, the samples were removed from the substrate using CNC wire electric discharge machining (WEDM). Standard procedures (metallographic) were used to polish the samples before they were etched with Kroll’s reagent solution (H_2_O:HNO_3_:HF:: 5:4:1) and HCl:FeCl_3_:H_2_O:: 5:1:20. An optical microscope (LOMO “Metam 31-LV”, Saint Petersburg, Russia) captured a macro-scale panoramic image of the fabricated joint. Phase analysis was conducted using an X-ray diffractometer (BRUKER-D8, Stuttgart, Germany) with a step size of 0.02 degrees and a dwell length of 0.5 s at Saint Petersburg State University, Russia. Microstructural and compositional studies were carried out through an Energy Dispersive Spectroscopy (EDS)-equipped scanning electron microscope (S-4800 Hitachi—Buckinghamsire, UK, Carl Zeiss “MerlinTM”—Oberkochen, Germany) at Saint Petersburg State University, Russia. Micro hardness measurements were performed using a Vickers micro hardness tester (Walter Uhl-VMHT002—Asslar, Germany) with a 1.9 N load and a dwell time of 10 s (as per ASTM E384-17) [37]. Small samples (3 × 3 mm^2^) extracted from the NiTi side’s edge, middle, and joint regions underwent martensitic transformation studies using Differential Scanning Calorimetry (Mettler Toledo 822—Malvern, United Kingdom) at Taltech, Estonia. In the temperature range of 100 °C to 110 °C, samples were subjected to heating and cooling at a rate of 10 °C/min. Uniaxial compression tests were conducted on samples with a width of 3 mm and a length of 6 mm (as per ASTM E9 [38]) using an INSTRON 1342 machine—Darmstadt, Germany with a 100 kN load cell at Taltech, Estonia. The loading speed was maintained at a constant of 0.5 mm/min.

## 3. Results and Discussion

### 3.1. SEM and XRD Results

**Figure 2:**
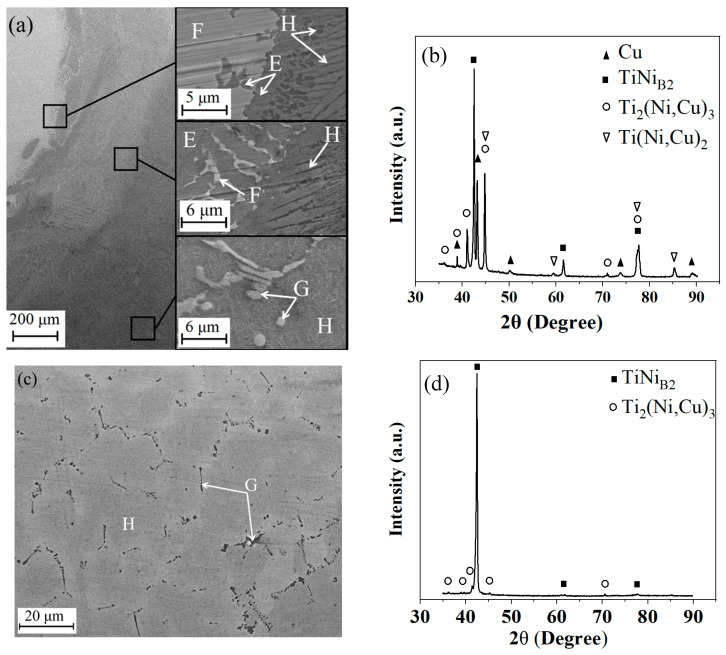
SEM images (**a**,**c**) and X-ray patterns (**b**,**d**) found in the vicinity of the joint (**a**,**b**) or far from the joint (**c**,**d**) in the NiTi layer of the NiTi/Cu bimetallic junction.

Figure 2 provides a detailed exploration of the SEM images and X-ray findings obtained from various points along the NiTi/Cu junction, offering valuable insights into its structural composition. Notably, the observation of an indistinct joint line between Cu and NiTi unveils a wide mixed zone spanning approximately 500 µm. Within this zone, a diverse array of constituents is observed, including the Ti (Ni,Cu)_2_ phase (designated as “E”), pure Cu inclusions (marked as “F”), Ti_2_ (Ni,Cu)_3_ precipitates (labeled “G”), and Ni-rich NiTi precipitates within the NiTi (Cu) phase (identified as “H”) (Figure 2a). The confirmation of the joint’s structural integrity is further reinforced by an analysis of the X-ray patterns (Figure 2b). Upon closer examination, a discernible reduction in the presence of Ti(Ni,Cu)_2_ and Ti_2_(Ni,Cu)_3_ precipitates is noted as one moves away from the joint towards the NiTi side. Conversely, the proportion of the NiTi(Cu) phase gradually increases in regions farther from the joint. In the NiTi layers, predominantly comprising the NiTi(Cu) phase, only minimal Ti_2_(Ni,Cu) precipitates are observed along the NiTi(Cu) grain boundaries (Figure 2c). Notably, the Cu concentration within the NiTi phase exhibits a decrease from 15 at. % near the joint to 1% in the uppermost NiTi layer. Despite positional variations, the combined Ni+Cu concentration within the NiTi phase consistently surpasses 50 at. %, with Ni concentrations ranging from 51 to 52 at. %, even within the upper layers. The X-ray analysis (Figure 2d) corroborates the presence of an austenite structure in the NiTi phase at room temperature, in line with the chemical composition detected through EDS analysis.

### 3.2. DSC Result

**Figure 3:**
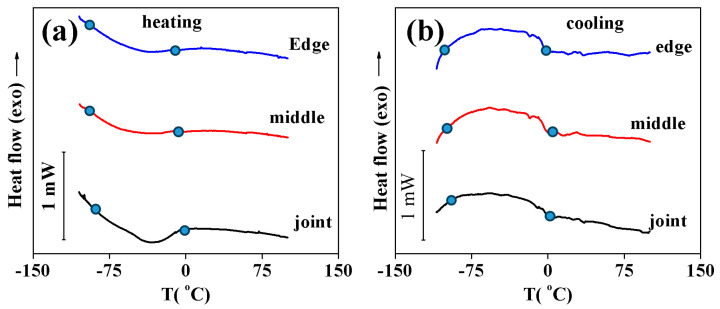
Calorimetry curves obtained upon cooling (**a**) and heating (**b**) samples cut from various location of the NiTi layers in NiTi/Cu bimetallic junction.

Figure 3 shows the calorimetric curves measured in the samples cut from three locations in the cross-section of the NiTi/Cu bimetal junction. In NiTi shape memory alloys, B2 is the high-temperature cubic phase, B19 is the low-temperature martensitic phase, and R is the rhombohedral phase during transformation. Low-intensity broad peaks were found upon cooling and heating. The transformations parameters (temperatures and enthalpy) hardly depended on the location of the samples in the NiTi/Cu junction and were characterized by small enthalpy and hysteresis (H = A_f_ − M_s_) that corresponded to the B2 → R transformations. According to SEM and X-ray analysis, a large number of the NiTi layers were occupied by secondary phases (Ti_2_(Ni,Cu)_3_ and Ti(Ni,Cu)_3_), which did not undergo martensitic transformations; that is why the volume fraction of the NiTi phase was small. This was one of the reasons for the observation of a low-intensive calorimetric peak. The other reason was a high Ni concentration in the NiTi phase, which decreased the transformation enthalpy. Transformation temperature ranges (Ms-Mf and Af-As) were very wide (~100 °C); this was due to the inhomogeneity of the chemical composition of the NiTi phase. As a result, various volumes of the NiTi phase underwent martensitic transformation at different temperatures, which led to the widening of the transformation temperature range [34,35,36].

**Figure 4:**
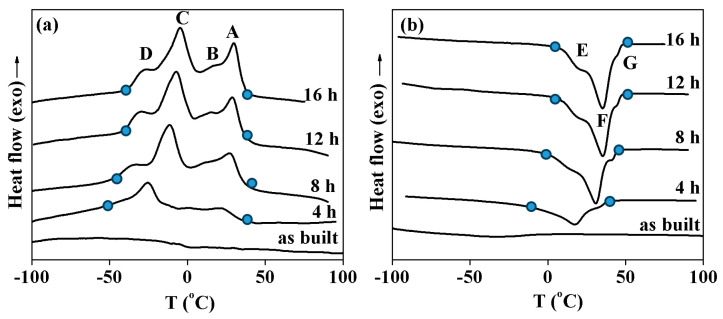
Calorimetry curves obtained upon cooling (**a**) and heating (**b**) samples cut from the NiTi layers near the joint in NiTi/Cu bimetallic junction. Annealing durations are shown near the curves.

The low transformation enthalpy and wide temperature range show that the samples are not able to demonstrate good functional properties. Thus, the martensitic transformation should be improved by heat treatment. To study the influence of heat treatment on the martensitic transformation, the samples were annealed at 500 °C for 4–16 h. Figure 4 shows the calorimetric curves measured in the samples cut near the joint after different heat treatments. It can be seen that annealing for 4 h at 500 °C leads to the observation of four peaks on cooling and three peaks upon heating. Using this special procedure, it was found that, upon cooling, peak A was caused by the B2 → R transformation and peak C corresponded to the R → B19′ transformation. On heating, this B19′ phase transformed to a B2 phase within peak F. The peaks B and G were due to the B2 ↔ B19′ transformations; peaks D and E were caused by the same B2 ↔ B19′ transformation. The observation of different sequences of the transformation was due to the NiTi layer near the joint having various distributions of the Ti and Ni concentrations before annealing. This resulted in the annealing affecting, in various ways, the areas of the NiTi phase with different chemical compositions. In Figure 4, it can be seen that an increase in annealing duration increases the intensity and temperatures of all peaks. This was due to the Ni concentration in the NiTi phase decreasing during annealing, which increases the transformation temperatures and the volume fraction of the alloy undergoing the phase transformation. At the same time, a comparison of the calorimetric curves obtained after 12 and 16 h does not show any distinctions in transformation enthalpy or temperatures.

**Figure 5:**
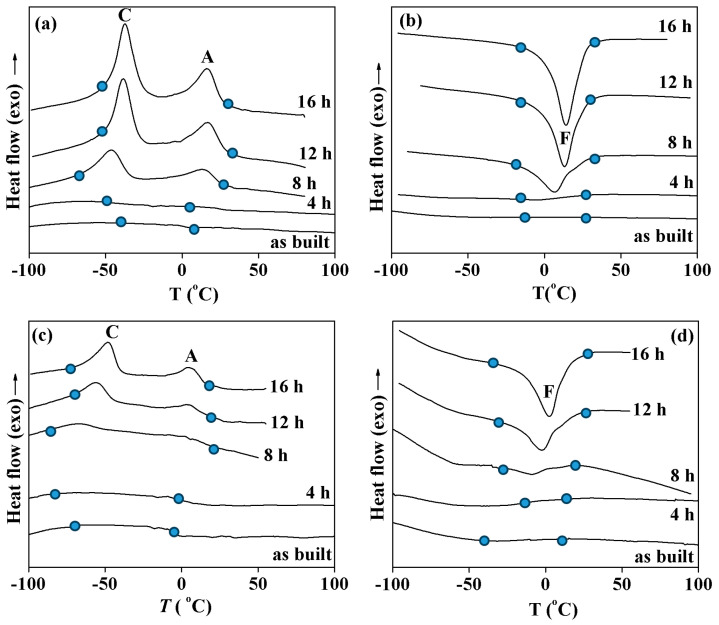
Calorimetry curves obtained upon cooling (**a**,**c**) and heating (**b**,**d**) samples cut from middle (**a**,**b**) or edge areas in the NiTi layers of the NiTi/Cu bimetallic junction. Annealing durations are shown near the curves.

Figure 5 provides a detailed depiction of the calorimetric profiles obtained from samples extracted from both the central and peripheral regions of the NiTi/Cu interface, after annealing at 500 °C for varying durations. In the comprehensive analysis, it becomes evident that, in both sets of samples originating from the central and peripheral regions of the NiTi layer, a discernible B2 → R → B19′ transformation occurs during the cooling phase (peaks A and C), succeeded by a B19′ → B2 transition upon heating (peak F). This transformational behavior underscores the dynamic nature of the material under thermal treatment. Remarkably, the annealing process exerts a more pronounced effect on the central regions, as indicated by the heightened intensity of the peaks observed in samples derived from the NiTi layer’s midpoint after 8 h of annealing, compared to those from the edge. This observed difference in transformation intensity may stem from the elevated Cu concentration in the central sample. The heightened Cu concentration facilitates precipitate formation during annealing, thereby reducing the Ni concentration within the NiTi matrix and augmenting the volume fraction of the alloy undergoing martensitic transformation.

Further analysis reveals that extending the annealing duration to 12 h amplifies the transformation enthalpy, indicating a deeper structural change within the material. However, durations exceeding 12 h yield negligible alterations in transformation parameters such as enthalpy and temperatures. This observation suggests a saturation point in the annealing process, beyond which additional time does not significantly influence the material’s transformational behavior. Consequently, it can be inferred that annealing at 500 °C for 12 h emerges as the optimal heat treatment regimen for effectively reinstating transformation within the NiTi/Cu interface. This finding underscores the importance of precise annealing protocols in controlling the material’s microstructural evolution and functional properties, particularly in intricate systems like the NiTi/Cu junction. 

### 3.3. Hardness and Compression Tests

**Figure 6:**
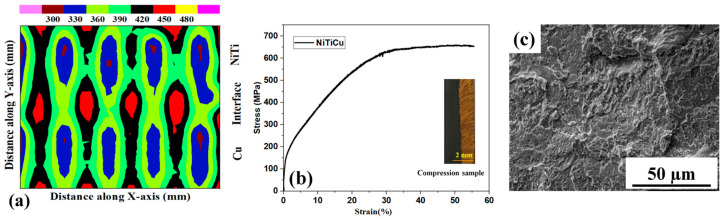
Mechanical behaviour of bimetallic joint in terms of (**a**) micro-hardness, (**b**) compression strength, and (**c**) fractography.

Figure 6a depicts comprehensive hardness results for the NiTi–Cu joint and base material, showing an average micro-hardness of 485 HV. Employing a spatially distributed strategy, variations were measured at intervals of 1.5 mm (Y direction) and 0.5 mm (X direction). The increase in NiTi micro-hardness results from copper incorporation, causing precipitation in the α-Ti phase due to the solid solution hardening of Cu in the alloy matrix [33]. Internal stress-induced rise is driven by precipitates like Ti(NiCu)_2_, particularly at higher Cu concentrations [34]. Elevated micro-hardness and embrittlement stem from Cu amalgamation in the NiTi region. Enhanced micro-hardness in the samples with over 10% Cu underscores the inherent brittleness in Cu-based intermetallic compounds [39]. In Figure 6a, the bottom part shows the hardness for pure Cu (100%), the middle depicts 15% Cu in the interface with Ni (45%), and the top shows NiTi hardness with 1% Cu, 51% Ni, and 48% Ti. This shows how the hardness values correlate with phase or composition in different NiTi–Cu joint regions. Figure 6b shows the compressive results for the interface (NiTi–Cu) and NiTi. The compressive strength (650 MPa) decreases after Cu is added to NiTi due to the production of brittle intermetallics. The elasticity and ultimate strength were at their highest at the lowest percentage of Cu, and they steadily decreased as the Cu weight percentage increased [33,34,35,36]. The documented mechanical outcomes surpass those achieved through traditional NiTi–Cu joint fabrication [35], indicating superior joint strength when compared to alternative methods. Figure 6c illustrates the fractured surface of the welded joints. Fractures occur along the cross-section of the welded joint. A brittle-like failure was observed in the fusion zone and ductile features were observed along the soft copper base metal. Upon closer examination using SEM, additional features became apparent: in the NiTi-Cu weld, the fracture region displayed smooth step patterns with cleavage-like features (Figure 6c). Notably, failure transpired through the fusion zone rather than along the formed interface because the intermetallic embrittled fusion zone could not withstand the load. This degradation stems from microstructural alterations induced by the WAAM deposition process, particularly attributable to Cu-based intermetallic compounds. These compounds fail to enhance the superelastic behavior, thus contributing to the decline in the mechanical properties of the constructed joints [34,35,36,39].

## 4. Conclusions

The microstructural analysis conducted on defect-free NiTi–Cu bimetallic structures provides valuable insights into the intricate solidification processes observed in both the NiTi and Cu sides. Dendritic solidification patterns characterize the formation of these structures, with discernible variations in Ti(Ni, Cu)_2_ and Ti_2_(Ni, Cu)_3_ precipitates within the NiTi layers. Interestingly, there is a noticeable decrease in Cu concentration as one moves away from the joint region. Despite the heterogeneity in composition, NiTi phases consistently exhibit austenite structures, which align with the chemical composition findings. However, the results from differential scanning calorimetry (DSC) pose challenges in achieving the desired functional properties, as evidenced by the presence of low-intensity broad peaks. The broad temperature range and low enthalpy underscore the necessity for enhancing martensitic transformation, a need addressed through a meticulous 12 h heat treatment at 500 °C. It is worth noting that Cu-rich regions display intensified transformations, highlighting the pivotal role of chemical composition in shaping the material’s behavior. Mechanical assessments further unveil an increase in micro-hardness within NiTi–Cu samples, attributed to Cu-induced precipitation and solid solution hardening. However, the introduction of Cu leads to a reduction in compression strength, primarily due to the formation of brittle intermetallics, as evident in detailed fractographic analyses. These observations underscore the significant microstructural changes that occur during the fabrication process.

In conclusion, this study significantly enhances our understanding of NiTi–Cu bimetallic joints, laying the groundwork for diverse applications across various fields. However, further exploration is imperative to delve into the thermo-mechanical response and transformation properties of NiTi/Cu joints under stress-loaded conditions. These joints serve as crucial connectors for pseudoplastic actuators within electromechanical systems, offering convenient electrical connections that are especially beneficial for SMA actuators characterized by non-standard geometries that impede mechanical crimping. The innovative connecting technique presented here holds immense promise for revolutionizing SMA applications and augmenting existing methodologies in the field.

## Figures and Tables

**Table 1:** Optimized parameters for the WAAM deposition.

Material	Wire Feed Rate (m/min)	Argon Gas Flow Rate (L/min)	Voltage (V)	Stand off Distance (mm)
NiTi	5	20	15	20
Cu			16.5	

## Data Availability

The data are part of ongoing studies and can be made available on reasonable request to the corresponding author.
